# *In Vitro* Functional and Structural Characterization of A Synthetic Clinical Pulmonary Surfactant with Enhanced Resistance to Inhibition

**DOI:** 10.1038/s41598-020-58248-4

**Published:** 2020-01-28

**Authors:** Mercedes Echaide, Chiara Autilio, Elena López-Rodríguez, Antonio Cruz, Jesús Pérez-Gil

**Affiliations:** 1 Department of Biochemistry and Molecular Biology, Faculty of Biology, and Research Institute “Hospital 12 de Octubre (imas12)”, Universidad Complutense, 28040 Madrid, Spain; 2 Present Address: Charité-Universitätsmedizin Berlin, Institut für funktionelle Anatomie, Campus Mitte, Philippstrasse 12, 10115 Berlin, Germany

**Keywords:** Membrane structure and assembly, Phospholipids

## Abstract

CHF5633 is a novel synthetic clinical pulmonary surfactant preparation composed by two phospholipid species, dipalmitoyl phosphatidylcholine (DPPC) and palmitoyloleoyl phosphatidylglycerol (POPG), and synthetic analogues of the hydrophobic surfactant proteins SP-B and SP-C. In this study, the interfacial properties of CHF5633 in the absence and in the presence of inhibitory serum proteins have been assessed in comparison with a native surfactant purified from porcine lungs and with poractant alpha, a widely used clinical surfactant preparation. The study of the spreading properties of CHF5633 in a Wilhelmy balance, its ability to adsorb and accumulate at air-liquid interfaces as revealed by a multiwell fluorescence assay, and its dynamic behavior under breathing-like compression-expansion cycling in a Captive Bubble Surfactometer (CBS), all revealed that CHF5633 exhibits a good behavior to reduce and sustain surface tensions to values below 5 mN/m. CHF5633 shows somehow slower initial interfacial adsorption than native surfactant or poractant alpha, but a better resistance to inhibition by serum proteins than the animal-derived clinical surfactant, comparable to that of the full native surfactant complex. Interfacial CHF5633 films formed in a Langmuir-Blodgett balance coupled with epifluorescence microscopy revealed similar propensity to segregate condensed lipid domains under compression than films made by native porcine surfactant or poractant alpha. This ability of CHF5633 to segregate condensed lipid phases can be related with a marked thermotropic transition from ordered to disordered membrane phases as exhibited by differential scanning calorimetry (DSC) of CHF5633 suspensions, occurring at similar temperatures but with higher associated enthalpy than that shown by poractant alpha. The good interfacial behavior of CHF5633 tested under physiologically meaningful conditions *in vitro* and its higher resistance to inactivation by serum proteins, together with its standardized and well-defined composition, makes it a particularly useful therapeutic preparation to be applied in situations associated with lung inflammation and edema, alone or in combined strategies to exploit surfactant-facilitated drug delivery.

## Introduction

The presence of a surface-active lipid/protein complex at the respiratory air-liquid interface is essential to facilitate effortless breathing mechanics^1,2^. Lack or dysfunction of this pulmonary surfactant is associated with severe, often lethal, respiratory pathologies^3^. Babies born before their lungs have matured to produce surfactant are at risk of developing respiratory distress syndrome (RDS), with a high mortality unless these neonates are treated early with an exogenous surfactant material. In this sense, administration of a bolus of exogenous surfactant, typically obtained from extracts of animal-derived materials, has saved thousands of preterm baby lives^4,5^. In other pathologies associated with lung injury and inflammation in children and adults, blood proteins and inflammatory mediators liberated into the airways as a consequence of edema and injury may inactivate surfactant function leading to lung atelectasis and respiratory failure^6,7^. Restoring surfactant activity upon administration of exogenous materials to these patients, has been so far prevented. In this line, enough amounts of clinical surfactants with well-defined composition and resistance to inactivation are required to treat adult injured lungs.

CHF5633 (CHF) is a synthetic surfactant, which has completed a phase I clinical trial^8^ and is currently under phase II. It is a simple mixture which contains 98.3% lipids by mass in a 1:1 ratio of dipalmitoyl phosphatidylcholine (DPPC):palmitoyloleoyl phosphatidylglycerol (POPG), and 1.7% by mass of analogues for both hydrophobic pulmonary surfactant proteins, 0.2% of an analogue of SP-B and 1.5% of an analogue of SP-C^9,10^. Most effective surfactants used nowadays to treat neonatal Respiratory Distress Syndrome (nRDS), also partially effective in some cases of Neonatal and Adult Acute Respiratory Distress Syndrome (ARDS), are from natural origin, typically organic extracts of either calf or porcine bronchoalveolar lavages or from minced lungs^5^. However, materials obtained from animal sources could present some potential inconveniences, such as a) the impossibility to have a constant and repetitive formula, b) the possibility of an immunogenic response if many repeated doses are required, and c) the amount of animals needed to obtain the product with a high cost of production. Although the effective appropriate dosage to treat adults under pathological situations is not well defined, higher amounts of surfactant are needed for each dose, and more than one dose is usually necessary for the treatments. On the other hand, nowadays, surfactant is starting to be considered as a possible carrier for inhaled drugs to treat either respiratory pathologies or other diseases, with more focused targets and less side effects. To do so, a simple surfactant with good spreading properties could be enough to allow medicines reaching the deepest alveoli, but it is anticipated that its use as drug carrier will open the need for much larger amounts of clinical surfactants with well established composition and performance.

Previous efforts have been done to obtain a synthetic surfactant as simple as possible but with a proper biophysical performance to treat patients (revised in^7^). First attempts included only lipids but once it was established that hydrophobic proteins are crucial for the biophysical function of surfactant, synthetic mixtures incorporated peptides mimicking proteins SP-B and/or SP-C^11^. Later studies showed that a better performance, both *in vitro* and *in vivo*, was obtained when analogues of both proteins were included into the lipid/peptide formulation^12^.

In the present study, we have performed a detailed *in vitro* biophysical characterization of CHF. The SP-B analogue into CHF is a 34-amino acid peptide derived from the two halves (residues 8–25 and 63–78) of the human SP-B, with two intramolecular disulphide bridges mimicking disulphides in the native protein. The SP-C analogue in CHF is a 33-amino acid peptide resembling native SP-C, where valines have been substituted by leucines giving rise to a more stable protein. The properties of CHF have been compared with a worldwide effective clinical surfactant from natural origin, namely poractant alfa (PORα) (Curosurf ®, Chiesi Farmaceutici S.p.A., Parma, Italy), and with native porcine native surfactant (NS) obtained from bronchoalveolar lavage. Particular attention has been paid to the biophysical behaviour of the surfactants in the presence of serum or albumin, well-established inhibitors of surfactant performance^13^.

## Results

### Interfacial behaviour

**Figure 1:**
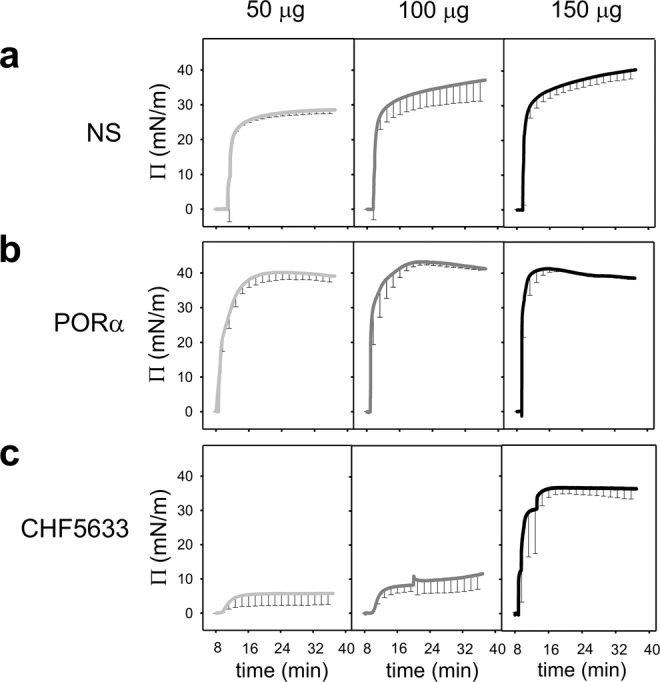
Adsorption isotherms of surfactants in a Wilhelmy balance. Spreading isotherms of 50 (light grey), 100 (dark grey) and 150 μg (black) of (**a**) NS, (**b**) PORα and (**c**) CHF. Surfactant was applied in a single drop at the air-liquid interface. Surface pressure was measured along 40 minutes. The mean of three replicates is depicted. Black vertical lines represent standard deviations. Abbreviations: NS = porcine Native Surfactant, PORα = Poractant alfa (Curosurf ®).

Figure 1a–c shows the kinetics of spreading over the interface for the three tested surfactants at 25 °C, when applying 50 μg, 100 μg or 150 μg of NS, PORα or CHF respectively. While NS and PORα are able to rapidly spread over and adsorb into the interface even when small amounts of material are applied, CHF needs higher amounts of surfactant at the interface to reach a surface pressure close to that of equilibrium. Moreover, the equilibrium is reached by CHF at a somehow slower rate, with a jump in surface pressure typically occurring after 10 min of sample deposition.

**Figure 2:**
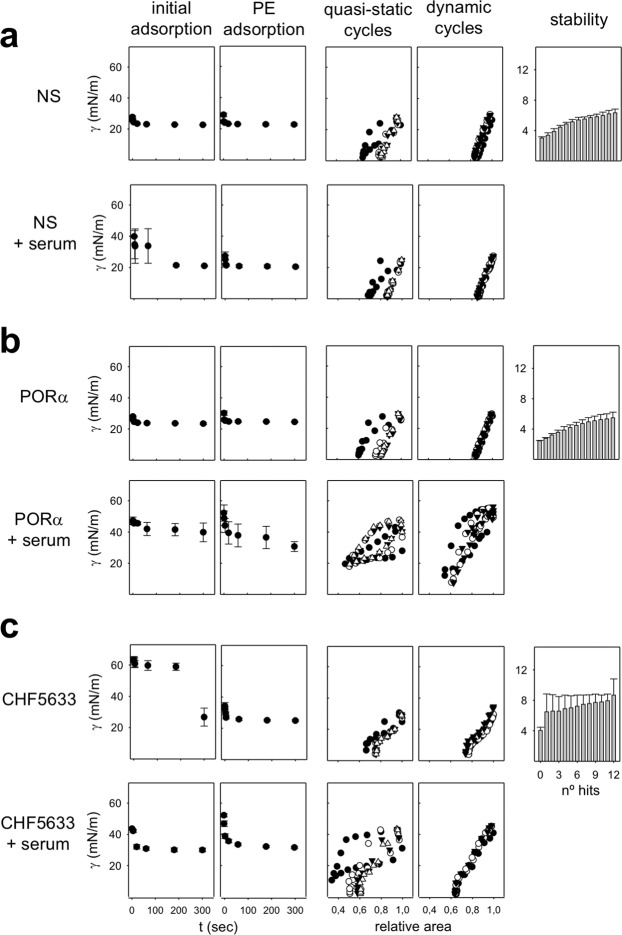
Interfacial behaviour of surfactants as assessed in a Captive Bubble Surfactometer. Initial and post-expansion adsorption and slow quasi-static and fast dynamic compression-expansion isotherms as obtained in CBS experiments are compared for (**a**) NS, (**b**) PORα and (**c**) CHF5633. Around 300 nL of surfactant at 10 mg/mL were injected onto the bubble in each experiment, in the presence or absence of 3 µL of porcine serum. *Initial adsorption, Post-expansion (PE) adsorption and stability:* the mean of three replicates is depicted. Black circles and grey bars represent surface tension. Black vertical lines represent standard deviations. *Quasi static cycles:* a representative experiment is shown. Changes in surface tension during the 1^st^ (black circles), 2^nd^ (white circles), 3^rd^ (black triangles) and 4^th^ (white triangles) compression-expansion cycles are depicted. *Dynamic cycles:* a representative experiment is shown. Changes in surface tension during the 1^st^ (black circles), 10^th^ (white circles) and 20^th^ (black triangles) compression-expansion cycles are depicted. Abbreviations: NS = porcine Native Surfactant, PORα = Poractant alfa (Curosurf ®).

Formation and performance of surface films under more physiological conditions were assessed in the Captive Bubble Surfactometer (CBS). Figure 2 summarizes the surface behaviour of the different surfactants tested in this device. Samples are applied onto the surface of a small (5 mm) air bubble enclosed in a thermostated (37 °C) chamber and subjected to compression-expansion cycling mimicking breathing dynamics. The initial adsorption kinetics of NS, PORα and CHF is shown in the first panels of Fig. 2, in the absence (first, third and 5^th^ rows) or presence (2^nd^, fourth and sixth rows) of a layer of serum. In the absence of serum, NS and PORα reach equilibrium values (∼23 mN/m) after only 1 second from the injection of material. Conversely, CHF presents a relatively slower initial adsorption at the concentration tested (10 mg/ml.) This material needs around 3 min to reduce surface tension to values around 26 mN/m. Interestingly, upon increasing the number of replicates, the adsorption isotherms of CHF showed a high experimental variability with the surfactant adsorbing in some experiments as fast as NS or PORα, indicating a non-homogenous initial adsorption (data not shown).

As the bubble is expanded and a new interface is opened, surfactant accumulated close to the interface was rapidly adsorbed achieving in a few seconds the equilibrium surface tension values in all the materials tested (Fig. 2, PE adsorption in the absence of serum). CHF, which exhibited slower initial adsorption than NS or PORα, had comparable fast post-expansion adsorption, indicating that once properly connected with the interface, CHF was able to efficiently promote transfer of surface active species into a newly open interface.

The analysis of γ-relative area isotherms during slow quasi-static (QS) or rapid dynamic (Dyn) compression-expansion cycling revealed that NS and PORα films behave similarly, under both cycling conditions. Under QS cycling, films from both surfactants exhibit a reorganization of material at the interface during the 1^st^ cycle. They need a larger extent of compression to reach the lowest surface tensions, and present a larger hysteresis, than that observed in subsequent cycles. At the end of the 2^nd^ compression cycle, both materials reach a surface tension of ≤2 mN/m with less than 20% of area reduction. Maximal surface tension at the end of the expansion steps under QS conditions is slightly lower than 30 mN/m. Under dynamic cycling, at compression‐expansion rates comparable to breathing, both NS and PORα films exhibit practically no hysteresis and extreme stability, producing the lowest surface tensions (≤3 mN/m) along all the cycles with less than 20% area reduction. Maximal tension under dynamic conditions is slightly higher than in the QS regime, but still no much higher than 30 mN/m.

CHF films also work very well under both QS and dynamic cycling, although it shows a behaviour that is somehow different than the typical exhibited by NS and PORα. Under QS conditions, it already achieves minimal surface tensions from the 2^nd^ compression cycle, although this minimal value is slightly higher (∼4 mN/m) compared with NS and PORα (∼2 mN/m). However, in QS as well as in Dyn cycles, CHF needs slightly more than 20% area reduction to achieve the lowest surface tension values. Remarkably, CHF isotherms at the CBS exhibit a conspicuous plateau at 15–20 mN/m, both in the compression and the expansion moieties of the isotherms. This denotes that CHF films undergo some sort of structural transition before reaching the less compressible stages that are competent to reach the lowest surface tensions.

As shown also in Fig. 2, CHF films are less stable under mechanical perturbations than PORα or NS films. Normally, after the first hit of a pendulum hammer over the CBS chamber, surface tension in CHF-stabilized interfaces raises in a jump of 3 or 4 mN/m while this increase is slower in interfaces stabilized by the other materials. It is worth noting anyway, that there is also more variation between the behaviour of different CHF samples than that exhibited by PORα and NS samples.

### Serum inactivation

Surfactant inactivation by serum proteins was assayed in the CBS as it was previously optimized^14^, by directly applying serum onto the air bubble before surfactant injection (Fig. 2a–c). This way, serum proteins have the opportunity to adsorb to the interface before surfactant is applied. Synthetic CHF is more resistant to serum inactivation than PORα, as can be clearly observed in its dynamic behaviour, more similar to that of NS. Though it needs to be compressed to a somehow greater extent, CHF films are able to reach the lowest surface tension even at the 2^nd^ QS cycle, with no hysteresis during the Dyn cycles. These results are in accordance to what it was previously observed in an *in vivo* model of RDS, where bronchoalveolar lavages (BAL) from preterm lambs treated either with PORα or CHF were analysed in the CBS^15^.

**Figure 3:**
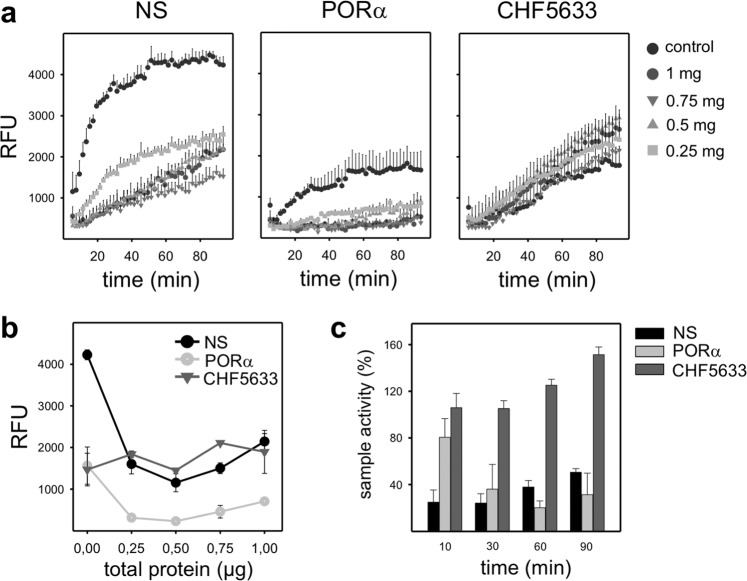
Interfacial adsorption and accumulation of surfactants. (**a**) Overtime adsorption kinetics and (**b**) surfactant performance after 60 min for NS, PORα and CHF in the presence of increasing amounts of total protein (mainly albumin) in the subphase. (**c**) Overtime surfactant activity when the highest amount of total protein is dispensed at the air-liquid interface. Circles and bars represent the mean of three replicates. Black vertical lines represent standard deviations. Abbreviations: NS = porcine Native Surfactant, PORα = Poractant alfa (Curosurf ®), RFU = Relative Fluorescence Units.

To confirm this apparent resistance of CHF to inactivation, a multiwell fluorescence assay (Surfactant Adsorption and Accumulation Test, SAAT) was used to measure the accumulation of NS, PORα and CHF at the interface in the absence or presence of different amounts of total serum proteins as described in Cerrada *et al*.^16^ (Fig. 3, panel a). Though CHF control presents a slower slope, it is surprisingly less altered by serum proteins than the other surfactants tested. In panel b of Fig. 3, surfactant accumulation at the surface after 60 min is shown. In the case of CHF, the presence of serum proteins in the subphase have almost no effect in the accumulation of material at the interface while PORα is quite affected even at the lowest amount of serum protein tested. As it could be expected, NS has a better performance in the absence of inactivating material, however, its relative activity is more affected than that of CHF when serum is present, as it is observed in the percentage of activity (panel c). Remarkably, NS and CHF increase their activity (accumulation at the surface) overtime, whereas PORα is more inactivated. In summary, it can be concluded that in the presence of serum, CHF is apparently as good as NS.

### Structural organization of surfactant films

Interfacial films formed by a pure 1:1 DPPC/POPG lipid mixture do not show phase segregation at 25 °C, at any surface pressure (not shown). The lack of domain segregation could be a consequence of the high proportion of unsaturated and charged phospholipid species.

**Figure 4:**
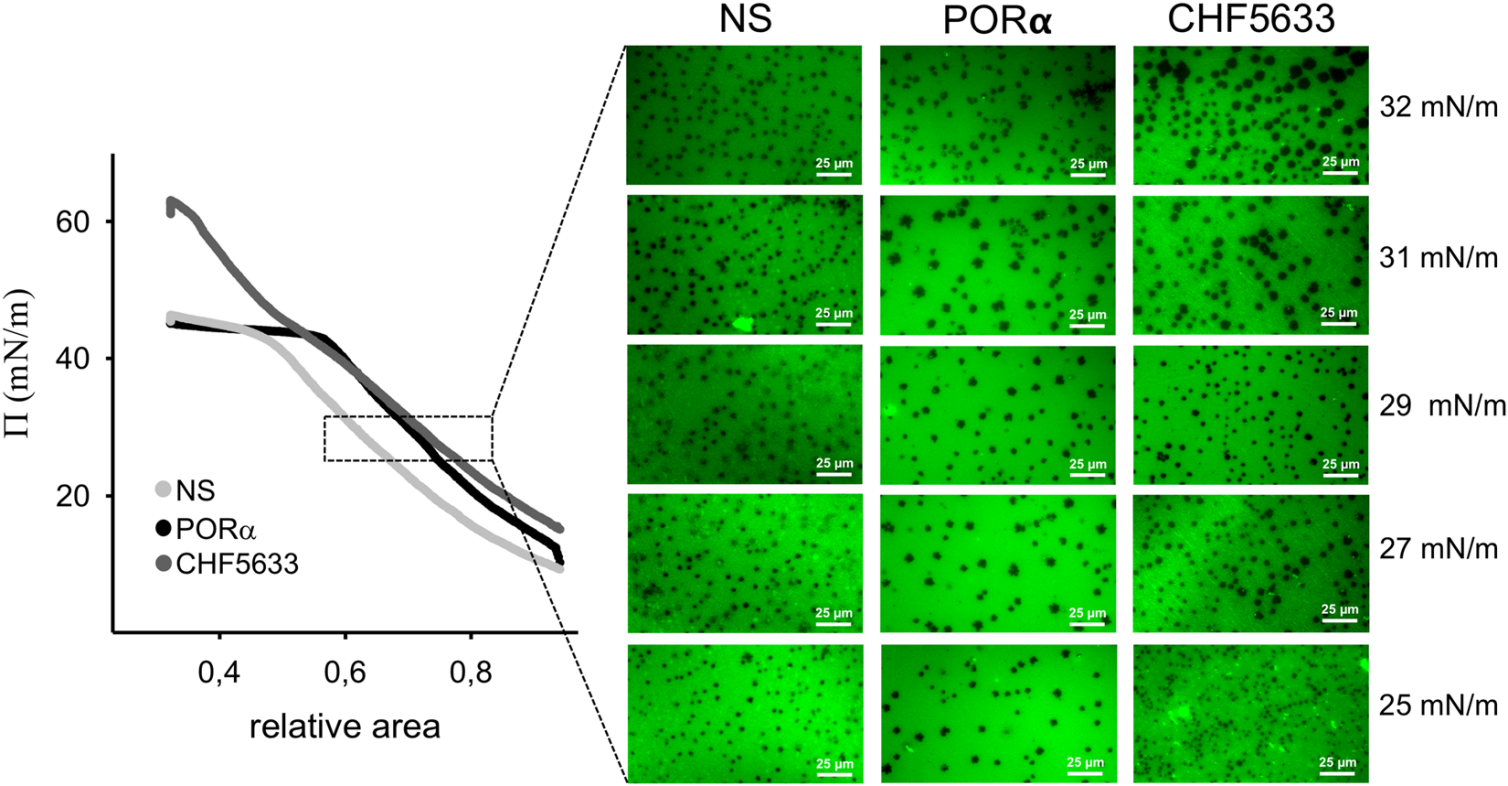
Compression-driven lateral structure of surfactant films. *Left*, a representative compression isotherm of films formed by each of the three surfactants tested is shown. 20 μL of NS and PORα at 2.5 μg/μL were applied at the air-liquid interface before starting compression. 3 µL of CHF5633 at 50 μg/μL were applied at the air-liquid interface, with the barrier completely closed before opening it to start compression. *Right*, lateral micro-structure of surfactant films (doped with 1% of NBD-PC) compressed to different surface pressures and observed under a epifluorescence microscope. Abbreviations: NS = porcine Native Surfactant, PORα = Poractant alfa (Curosurf ®).

In contrast, films formed by CHF suspensions show a very conspicuous phase segregation, once compressed to surface pressures above 25 mN/m, observed by the appearance of numerous small round dark domains, similar in size and shape to those observed in NS or PORα (Fig. 4). Also, it can be observed that CHF films present fluorescent bright spots where an accumulation of the label has been produced, at low surface pressures analysed before the surfactant saturates the interface.

### Thermotropic phase transitions

**Figure 5:**
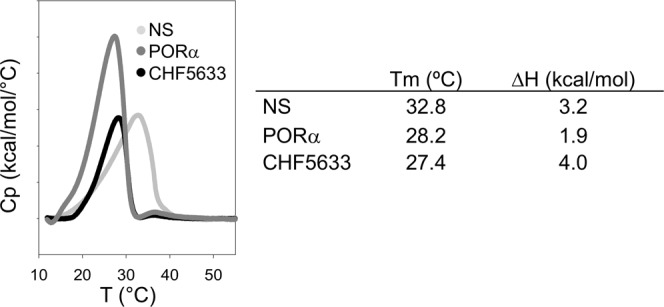
Thermotropic behaviour of surfactants by Differential Scanning Calorimetry. *Left*, a representative replicate of the DSC thermogram of the three surfactants tested is shown. 500μL of degassed material at 3 mg/mL were used. *Right*, main parameters from DSC thermograms. Abbreviations: NS = porcine Native Surfactant, PORα = Poractant alfa (Curosurf ®).

As previously published, NS membranes exhibit a melting temperature at around 32 °C, and a main calorimetric peak ending abruptly close to 37 °C with a typically broaden differential scanning calorimetry (DSC) thermogram^17^. Total enthalpy associated with the melting of ordered phases in NS is around 3 kcal/mol. PORα and CHF melt at significantly lower temperatures than NS, 28 °C and 27 °C respectively. PORα shows a relatively lower associated enthalpy, in the order of 2 Kcal/mol while thermotropic transitions occurring in CHF complexes liberate more enthalpy (∼4 Kcal/mol) than native surfactant or PORα (Fig. 5).

## Discussion

In this study, we have investigated on both the functional and structural features of a new synthetic clinical pulmonary surfactant, CHF. We have characterized its performance in comparison with native surfactant and PORα, one of the clinical surfactants from natural origin most used in replacement therapy of RDS^3,5^. The lipid-protein content and the structure of lung surfactant determine its three crucial biophysical properties: (1) a rapid adsorption towards the air-liquid interface, (2) an appropriate compressible behaviour while inhaling and exhaling to produce a minimal surface tension <5 mN/m at the end of exhalation, and (3) a good re-adsorption and re-spreading of material eliminated from the interface during the compression of the film upon inhalation^18^. In this line, the high percentage of DPPC and POPG in CHF, along with the only presence of the two hydrophobic peptides define its particular biophysical behaviour. However, for optimal interfacial adsorption of surfactant, not only the two hydrophobic proteins, SP-B and SP-C, but also the collectin SP-A play a key role. The latter seems very important to maintain a packed reservoir interconnected to the interface, thus contributing to the re-adsorption and expansion of new material during each compression cycle^19^. The presence of SP-A explains the best rates of both adsorption and accumulation at the interface of NS, compared with the SP-A-free clinical materials. In fact, PORα, derived from the organic extract of lung surfactant, from which SP-A is depleted, shows slower adsorption and accumulation rates (SAAT experiment). This adsorption difference between NS and PORα is not patent in the CBS isotherms, where surfactant is not applied in so limiting amounts. However, the lower adsorption rate of PORα is higher than that exhibited by CHF, in which not only SP-A is absent, but also the efficiency of synthetic peptides to transfer lipids into the interface may be reduced in comparison with the native proteins.

Moreover, as it happens for the adsorption, during spreading experiments, the application of low amounts of CHF at the interface was related with a worse initial performance of this material in the Wilhelmy balance. As argued above, we propose that this result is probably due to: (1) the higher content of DPPC −50% by mass compared to NS and PORα (in which DPPC accounts for around 40%^9^)- which makes the system less dynamic due to its saturated acyl chains, particularly when tested at 25 °C; (2) the nature (synthetic fragment vs native) and content (0.2% vs 1% in NS) of the SP-B analogue. However, the worse performance of CHF only emerges under limiting conditions and is absent when higher concentration and doses^20^ of material are applied. Similarly, to reach the equilibrium surface pressure during the Langmuir-Blodgett experiments, we needed to apply higher amounts of CHF, around two times more, at a higher concentration and in a smaller surface area, than that necessary for NS and PORα. In this line, elevated concentration of CHF in the CBS allows for a proper re-spreading as it is deduced from both the equilibrium tension values reached during post-expansion and the “low” maximal tension achieved upon cycling. CHF films exhibit in fact a very good dynamic performance: they reach low surface tension when compressed and not so high maximal values when the air bubble is expanded. We speculate that this good performance upon dynamic cycling may be associated with an almost complete exclusion of POPG from the interface during compression because of the unsaturated nature of such phospholipid species. Compression-driven POPG exclusion could be responsible for both the reproducible and reversible conspicuous plateau in the CHF isotherms, and the bright spots at the structure of the film observed under the epifluorescence microscope. Cooperative exclusion of POPG would leave a film mainly composed of DPPC at the air-liquid interface, thus contributing to a quick fall in surface tension to extremely low values. Excluded POPG would remain connected to the interface, presumably through the participation of surfactant peptides, allowing for its fully reversible reinsertion when the interface is re-opened during expansion. Typically, plateaus in the compression isotherms of surfactant films are associated with lateral or three-dimensional structural transitions preceding the acquisition of the non-compressible states required to reach the minimal surface tension (maximal lateral pressures). Often, such plateaus exhibit a variable degree of hysteresis, which is indicative of the work expended upon film transformation during compression^21^. The plateau in the CHF isotherm is clearly non‐hysteretic, which means that the structural transition producing the plateau is perfectly reversible and therefore could not be necessarily associated with a significant loss of energy. Moreover, it underlies that POPG is not merely lost during compression, but remains associated through the two peptides and thus providing a higher activity during the following re-extension.

Interestingly, not only the function but also the lateral structure of CHF films is similar to those of surfactants from natural origin^22^, something that is clearly linked to the presence of surfactant peptides. We may speculate that the presence of the proteins counteracts, at least in part, the negative charge of phospholipids facilitating lipid clustering and nucleation therefore triggering domain condensation^23^. Presence of protein analogues could therefore be particularly important in this system to facilitate the lateral sorting and domain segregation that favours a simultaneous flexibility and stability of the films. It remains to be established whether the two protein analogues have similar or distinct contribution to film structure. As mentioned above, CHF films reveal the presence of numerous fluorescent bright spots somehow associated to the condensed domains. We propose that these bright spots revealed as a consequence of light scattering under the epifluorescence microscopy are likely three dimensional surfactant aggregates attached to the surface film^23^, probably enriched in the less compressible species, presumably POPG. These spots could both act as a source of material to re-adsorb into the interface during expansion and to cooperate in a more efficacious exclusion of uncompressible lipids during compression.

The synthetic preparation shows melting at relatively low temperatures, but with higher associated enthalpy than PORα. This raised enthalpy could be related with the higher proportion of DPPC in CHF formulation compared with the other surfactant preparations, something that could be in principle associated with higher melting temperatures. However, the low Tm of CHF, around 27 °C, could be explained because of the remarkable high amount of POPG, which has a Tm of −2 °C. The thermogram of CHF complexes, as that of PORα, is more cooperative (less broaden) than that of native surfactant. The broaden transition exhibited by NS is likely related to the presence of cholesterol in NS membranes and its effect in segregating lipid populations with different packing and melting properties^17^.

An open question is whether surface‐associated aggregates in CHF are required for the good performance of this surfactant in the presence of inhibitory substances such as serum proteins. To get deeper into this topic, we studied the *in vitro* inhibition of the three surfactants in the presence of serum. In fact, during lung inflammation and altered epithelial permeability, as it occurs in cases of ARDS, serum is leaked into the alveolar spaces and, among its several components that may perturb surfactant membranes, serum proteins may directly inactivate surfactant function, as a consequence of a direct competition for the air-liquid interface^7,24^. The low compressibility of serum proteins does not allow for the extreme reduction in surface tensions that are required to prevent alveolar collapse. Other inflammation-associated proteins like CRP^25,26^ or secretory phospholipase A2^24,27^ also contribute, altogether, to inhibit surfactant performance. In our *in vitro* model, CBS results confirm a similar performance of CHF in the absence or presence of serum, including the possible exclusion of POPG that would be associated with the characteristic plateau of the isotherm during dynamic cycles.

As it could be expected, full native surfactant containing the originally assembled structures exhibits the highest resistance to inactivation, being able to displace serum proteins away from the interface when it is not at very limiting concentrations. On the contrary, PORα seems to exhibit the worst performance amongst the materials tested, being CHF more resistant, in agreement with what it has been already observed *in vivo*^15^. We also demonstrated that CHF is more resistant to inhibition than PORα by assessing the total accumulation of material at the interface in the presence of increasing amounts of total serum proteins. This is also noticeable in the CBS where, in the presence of serum, adsorption of PORα is worse and upon quasi-static compressions, surfactant is not able to displace serum proteins, in contrast to CHF. Surfacen, another natural clinical surfactant preparation derived from BAL of porcine lungs, shows the same kind of dynamic isotherms as PORα, with a poor behaviour in the presence of serum^28^. Conversely, CHF films, though needing a larger compression extent, have still no hysteretic isotherms when subjected to dynamic cycles in the presence of serum. We could speculate that there is a preferential exclusion of inactivating proteins that are swept along from the interphase together with POPG during the compression-expansion cycles. In fact, it is known that proteins such as fibrinogen, are excluded to the liquid-expanded phase while lateral compression^29^.

In summary, CHF5633 is a synthetic surfactant with great potential to be used in clinical therapies. Even if it does not adsorb at first instance as efficiently as other clinical surfactants from natural origin, it is capable of quickly spreading along the interface at clinical doses. Its synthetic and reproducible composition makes it a good candidate in the treatment of adults when high and multiple doses are needed, avoiding safety issues with the potential presence of pathogenic entities from animal origin, or possible allergenic reactions^30^. However, its maximal advantage is the stability of its dynamic properties even in the presence of serum, making it a potentially useful preparation in situations associated with inflammation and edema as well as for drug delivery therapies to patients with inflamed and injured lungs.

## Methods

### Materials

Native surfactant (NS) was purified from bronchoalveolar lavage (BAL) of porcine lungs obtained from the slaughterhouse as previously described^13^. BAL in ice cold Tris 5 mM pH 7 in 0.9% NaCl was centrifuged for 150 g during 10 minutes to remove cells, and the supernatant was centrifuged for 1 hour at 4 °C 100,000 g to pellet whole surfactant complexes. Pellets were resuspended in NaBr 16% NaCl 0.9% and pooled before being loaded onto a sodium bromide gradient centrifugation performed as previously described to remove potential blood contaminants. Phospholipid concentration was determined by phosphorus mineralization^31^. Clinical surfactants, PORα and CHF5633, were provided by Chiesi Farmaceutici S.p.A. (Parma, Italy).

### Interfacial surfactant performance

#### Wilhelmy balance

The spreading capability of surfactant was assayed by using a Wilhelmy balance whose design was modified by adding a small arm to the trough (total area arm = 6.45 cm2, total area trough = 21.45 cm2) to increase the distance between the injection point and the pressure sensor. NS, PORα and CHF were tested at 25 °C by spreading, directly at the air-liquid interface, 10 μL of the different materials containing 50, 100 or 150 μg of phospholipids. The sample was distributed only in one drop at the end of the smaller arm of the trough on the opposite side to the pressure sensor. Each experiment was performed in triplicate and the changes in surface pressure were recorded for 40 minutes.

#### Captive bubble surfactometry (CBS)

CBS permits to test surfactant activity in terms of the ability of surfactant samples to reach and maintain very low surface tension (γ) at the air-liquid interface of a small (5 mm) air bubble at a controlled temperature (37 °C), while mimicking the cyclic changes in alveolar volume that occur during breathing^32^. This device allows for the detailed analysis of several surfactant biophysical properties, including its ability to adsorb at the interface, to re-spread once new interface is open, to maintain low enough surface tension along quasi-static (slow) or rapid (breathing-like) dynamic compression-expansion interfacial cycling, or the mechanical stability of the films formed by the tested materials. Briefly, around 300 nL of each given surfactant suspension (at a concentration of 10 mg/mL) was injected close to the air-liquid interface generated between the air inside the microbubble and the buffer (5 mM Tris and 150 mM NaCl, pH 7, containing 10% sucrose to increase density and thus permitting surfactant floating). The changes in surface tension for 5 minutes after surfactant injection were quantified (Initial Adsorption) by continuously recording the variations in the bubble size and shape. Then, the chamber was sealed and the bubble was quickly expanded by decreasing the hydrostatic pressure generated by a piston. The capability of surfactant to re-extend and reduce again the equilibrium surface tension was monitored during further 5 min after bubble expansion (Post-Expansion Adsorption). The bubble was then sequentially subjected to both slow quasi-static (4 cycles) and quick dynamic (20 cycles/min) compression-expansion cycles. The changes in bubble size during cycles were video recorded, permitting the analysis of volume, area, and γ at any time. Finally, the stability of the multi-lamellar structure created by the interfacial surfactant films during dynamic cycling of the interface was tested by introducing shocking mechanical perturbations into the bubble chamber, hitting 12 times the chamber with a pendulum hammer, as previously described^32^. Experiments were performed in triplicate and results were analysed as γ/t and γ/hits graphs and γ/area isotherms.

Serum inhibition and surfactant resistance to this inhibition were tested as previously published^14^. In detail, 3 μL of whole porcine serum (at 67.9 mg/mL) was first injected at the air-liquid interface of the bubble in the CBS. After 5 minutes, surfactant was introduced and distributed over the bubble surface without touching the bubble, to initiate the experiment as described above from the Initial Adsorption but in the presence of serum.

#### Surfactant adsorption and accumulation test (SAAT)

The adsorption and accumulation of surfactant at the air-liquid interface were tested by SAAT as previously described^33,34^. In detail, 3 μg of surfactant were labelled with BODYPY-PC (Molecular Probes, Life Technologies, Carlsbad, California) for 1 hour at 37 °C to obtain a final molar ratio of 1% (dye/surfactant). This mixture (in a volume of 20 µL) was injected at the bottom of wells in a microplate whose wells were previously filled with 80 μL of a quenching solution (1% Brilliant Black, Sigma). To follow surface adsorption and accumulation, the fluorescence emission of the sample measured from the top was recorded overtime during shacking cycles of the microplate in a FLUOstar OPTIMA Microplate Reader (BMG Labtech, Offenburg, Germany). Fluorescence intensity reaching the surface was followed at 37 °C for 60 minutes (ie, for 30 readings). Experiments were performed in triplicate, and results are reported as relative fluorescence units (RFU). All data were corrected by subtracting the measured background.

Surfactant inhibition by total proteins was tested by adding to the Brilliant Black a volume of human serum containing different amounts of total protein (0.25, 0.5, 0.75 and 1 μg). Then, surfactant was injected and the experiment was assayed as described above.

### Surfactant structural properties

#### Epifluorescence microscopy

The lateral structure of interfacial surfactant films regarding the compression-driven segregation of liquid-condensed domains was observed by epifluorescence microscopy^21^. Briefly, 2.5 µg/µL of NS or PORα or 50 µg/µL of CHF were labelled with NBD-PC (Molecular Probes, Life Technologies, Carlsbad, California) for 1 hour at 37 °C to obtain a final molar ratio of 1% (dye/surfactant). In the case of NS and PORα, 15 µL of dye/surfactant suspension were spread at the air-liquid interface of a Langmuir-Blodgett trough (total area = 184 cm^2^, Nima Technology, Coventry UK). As for CHF, 3 µL of dye/surfactant suspension were applied with the barrier totally closed before opening it at a constant speed of 5 cm^2^/min. After 10 minutes equilibration, each surfactant film was subjected to compression at a constant speed of 25 cm^2^/min, while transferring the interface to a glass coverslip that had been previously immersed into the subphase. The resulting supported film, captured at different surface pressures as previously described^21^, was observed under an epifluorescence microscope (Leica microsystems, Wetzlar, Germany) equipped with a Hamamatsu digital camera.

#### Differential scanning calorimetry (DSC)

The thermotropic transitions between different phases in surfactant membranes and complexes were analysed by DSC as described^17^. Each surfactant was diluted to reach a concentration of 3 μg/μL in 5 mM Tris buffer pH 7, containing 150 mM NaCl. Then, after sample degassing, 500 μL of each suspension was loaded into the sample pan of a VP-DSC Microcal microcalorimeter, using the dilution buffer as reference. The differential heat (Cp) required to raise the temperature of the sample vs. reference was measured within a temperature range from 10 to 55 °C along 15 consecutive scans. Experiments were performed in triplicate, and results are reported as Cp/T functions.
